# Multimodality imaging analysis of the spectrum of endomyocardial fibrosis involvement in a clinical case series

**DOI:** 10.1093/ehjcr/ytaf039

**Published:** 2025-01-27

**Authors:** Josh Moscoso, Kevin Velarde-Acosta, Adriana Viñas, Lindsay Benites-Yshpilco, Angela Cachicatari-Beltran, Kelly Cupe-Chacalcaje, Luis Falcón-Quispe, Roberto Baltodano-Arellano

**Affiliations:** Cardiology Department, Hospital Guillermo Almenara Irigoyen—EsSalud, Av. Grau 800, La Victoria, Lima 15018, Peru; Cardiology Department, Hospital Guillermo Almenara Irigoyen—EsSalud, Av. Grau 800, La Victoria, Lima 15018, Peru; Cardiology Department, Hospital Guillermo Almenara Irigoyen—EsSalud, Av. Grau 800, La Victoria, Lima 15018, Peru; Cardiology Department, Hospital Guillermo Almenara Irigoyen—EsSalud, Av. Grau 800, La Victoria, Lima 15018, Peru; Cardiac Imaging Area of the Cardiology Department, Hospital Guillermo Almenara Irigoyen—EsSalud, Lima 15018, Peru; Cardiac Imaging Area of the Cardiology Department, Hospital Guillermo Almenara Irigoyen—EsSalud, Lima 15018, Peru; Cardiac Imaging Area of the Cardiology Department, Hospital Guillermo Almenara Irigoyen—EsSalud, Lima 15018, Peru; Cardiac Imaging Area of the Cardiology Department, Hospital Guillermo Almenara Irigoyen—EsSalud, Lima 15018, Peru; School of Medicine, Universidad Nacional Mayor de San Marcos, Lima 15018, Peru

**Keywords:** Endomyocardial fibrosis, Physiopathology, Restrictive heart failure, Multimodality imaging, Case series

## Abstract

**Background:**

Endomyocardial fibrosis (EMF) is a rare restrictive heart disease of the tropics. Its aetiology is cryptogenic; however, some hypotheses have been described. The clinical course is characterized by the presence of three phases (acute, subacute, and chronic). Most of the typical features of the disease are identified in the chronic phase. Multimodality imaging allows a presumptive diagnosis and cardiac magnetic resonance imaging, through its tissue characterization, allows confirmation of the diagnosis.

**Case summary:**

We present three clinical cases of patients with EMF, at different stages of the disease, which constituted a diagnostic challenge. However, it is due to multimodality imaging that a timely and accurate diagnosis is achieved.

**Discussion:**

Endomyocardial fibrosis is a rare heart disease with a restrictive phenotype and a poor prognosis. Endomyocardial fibrosis is characterized by apical obliteration with fibrous tissue, of one or both ventricles, and is usually associated with thrombosis, calcification, and AV valve insufficiency. Transthoracic echocardiogram is the first-line imaging modality to assess for EMF. Cardiac magnetic resonance, through late gadolinium enhancement, allows tissue characterization and identifies the ‘double V sign’, which is pathognomonic of the disease, allowing confirmation of the diagnosis. Therefore, multimodality imaging is essential for the initial and definitive diagnosis of this disease.

**Learning points**

- Endomyocardial fibrosis (EMF) is a rare heart disease with a restrictive phenotype and a poor prognosis.
- Multimodality imaging is essential for the initial and definitive diagnosis of this disease.
  - Transthoracic echocardiogram and transoesophageal echocardiography are the first-line imaging modalities to assess for EMF.
  - Cardiac computed tomography is valuable due to its high spatial resolution, attenuation-based tissue characterization, and functional assessment by the full-beat acquisition.
  - Cardiac magnetic resonance allows tissue characterization leading to confirmation of the diagnosis by identifying the ‘double V’ sign, which is pathognomonic of EMF.
  - The double V sign consists of a three-layered pattern of normal myocardium, endocardial thickening and fibrosis, and the presence of an overlying thrombus.

## Introduction

Endomyocardial fibrosis (EMF) is a rare restrictive heart disease of the tropics.^1^ Initially, the inflammatory process affects the ventricular apex and progresses to heart failure and thromboembolic phenomena.^2^ The first-line test is the transthoracic echocardiography, which provides key structural and functional information for detection.^3^ Cardiac computed tomography (CT) defines calcification; however, magnetic resonance imaging by using the late enhancement sequence identifies endocardial fibrosis and superimposed thrombi, which generates the pathognomonic ‘double V’ sign, allowing diagnosis.^4^ Multiple phenotypes of EMF may affect its diagnosis. A thorough understanding of the usefulness of multimodal imaging in this entity is necessary. To this end, we present three cases of EMF illustrated by multimodal imaging.

## Summary figure

**Figure:**
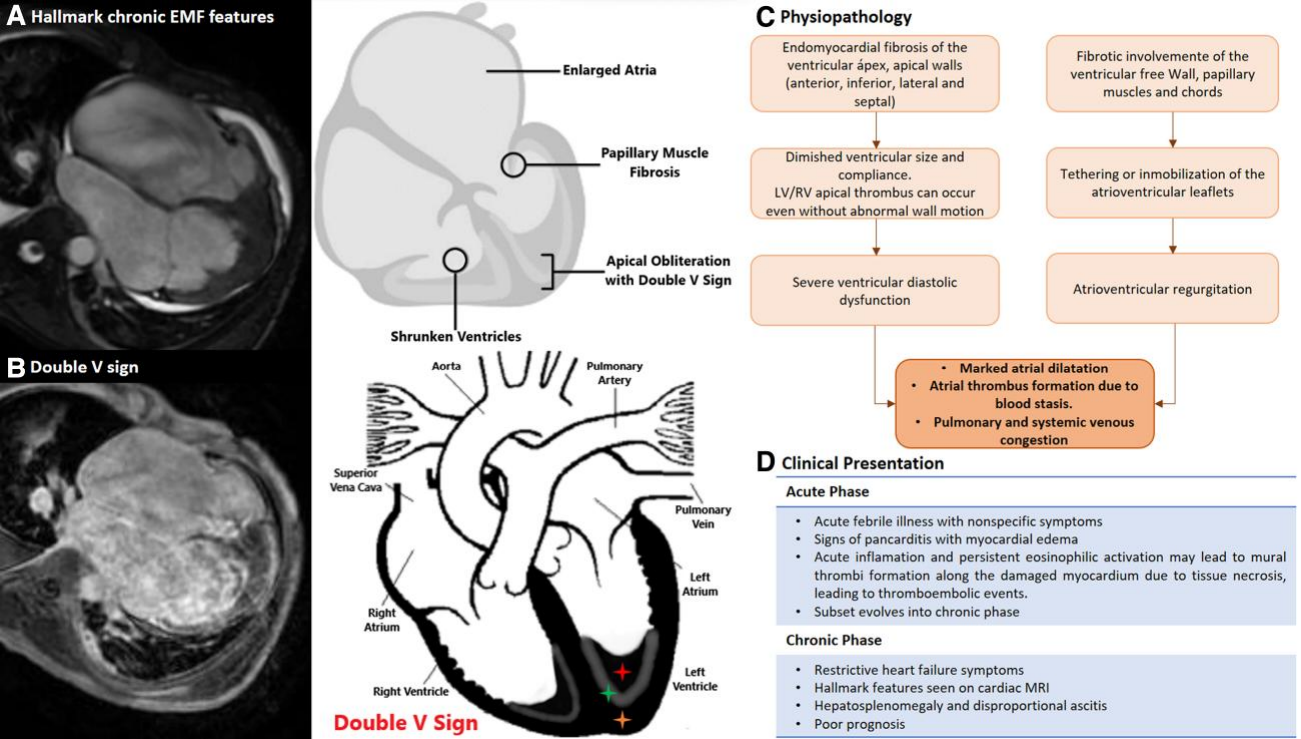
(*A*) Hallmark chronic endomyocardial fibrosis features. (*B*) Cardiac magnetic resonance pathognomonic sign: ‘double V sign’: a three-layered patter of normal myocardium (orange asterisk), endocardial thickening and fibrosis (green asterisk), and the presence of overlying thrombus (red asterisk) in the affected ventricular apex. (*C*) Endomyocardial fibrosis physiopathology. (*D*) Endomyocardial fibrosis clinical presentation. CMR, cardiac magnetic resonance; EMF, endomyocardial fibrosis.

## Case series

### Case 1

**Figure 1:**
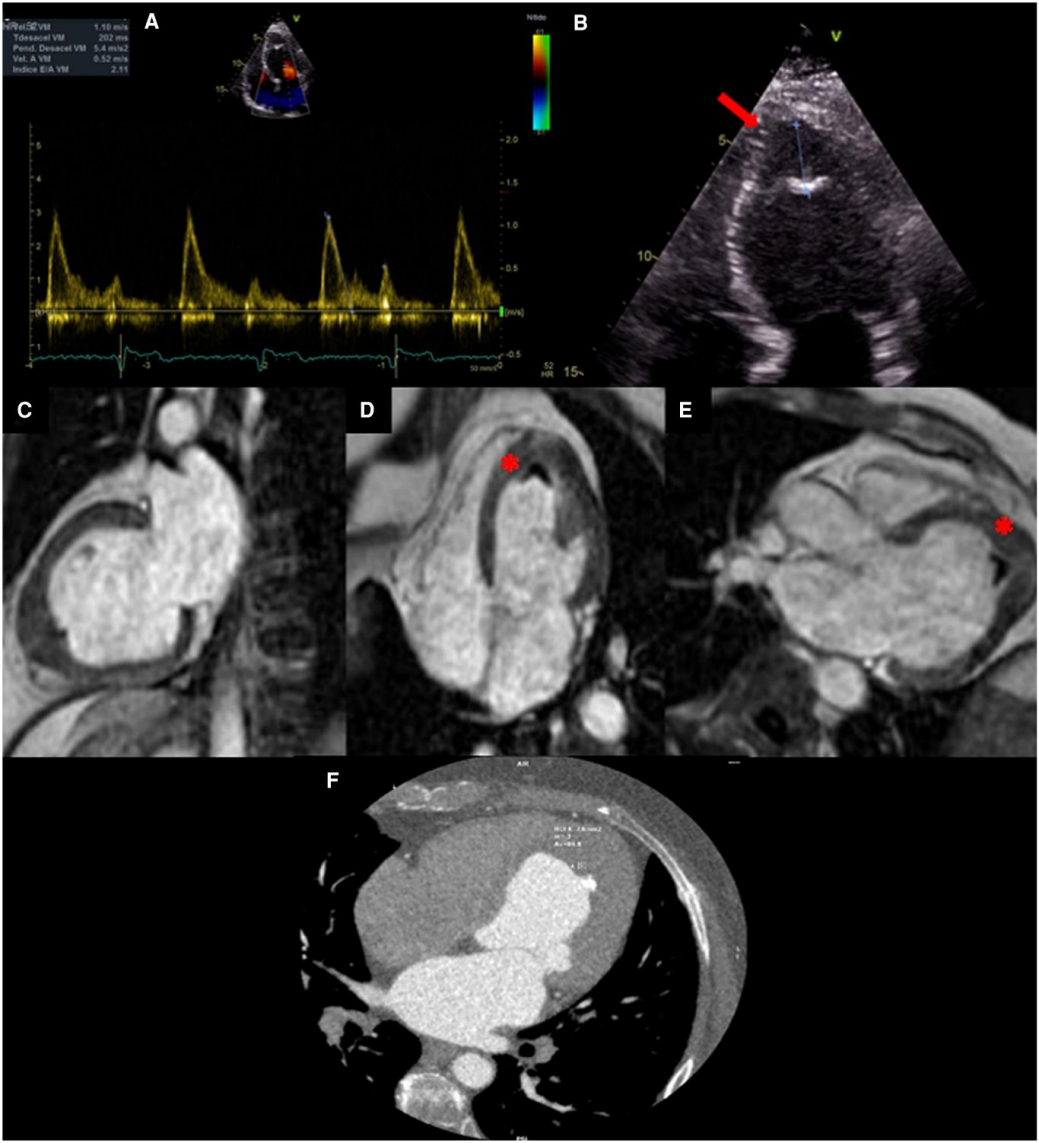
Case 1 images. (*A*) Pulsed Doppler demonstrates a restrictive filling pattern. Tissue Doppler showed a septal e′ wave at 5 cm/s. (*B*) Echocardiogram shows apical thickening with wedge-shaped internal calcification (arrow). In the lower row, we observe cardiac magnetic resonance in late gadolinium enhancement sequence. Two-chamber view (*C*) showing apical diastolic thickening and four- (*D*) and three-chamber views (*E*) showing apical endocardial enhancement with superimposed thrombus ‘double V sign’ (asterisk). (*F*) Cardiac computed tomography reformat four chambers showing apical obliteration with hypoattenuated image (85UH) and calcification.

A 42-year-old woman presented with palpitations. Relevant medical history included bronchial asthma and carpal tunnel syndrome. The patient was not receiving any medication on admission. Physical examination and laboratory findings were unremarkable. The electrocardiogram showed T-wave inversion in leads V2–6. Transthoracic echocardiogram revealed left ventricular (LV) apex thickening with an echodense mass suggestive of calcified mural thrombus (*Figure 1*; Supplementary material online, *Video S1*). She had no history of relevant infectious diseases. Apical hypertrophic cardiomyopathy (ApHCM) was initially suspected; however, cardiac magnetic resonance (CMR) showed LV apical obliteration (systolic–diastolic). The late gadolinium enhancement (LGE) sequence evidenced LV apex enhancement with the predominant involvement of the endocardial layer. Given the CMR findings, EMF was diagnosed. Conservative treatment and continuous monitoring were decided. Currently, symptoms have subsided.

### Case 2

**Figure 2:**
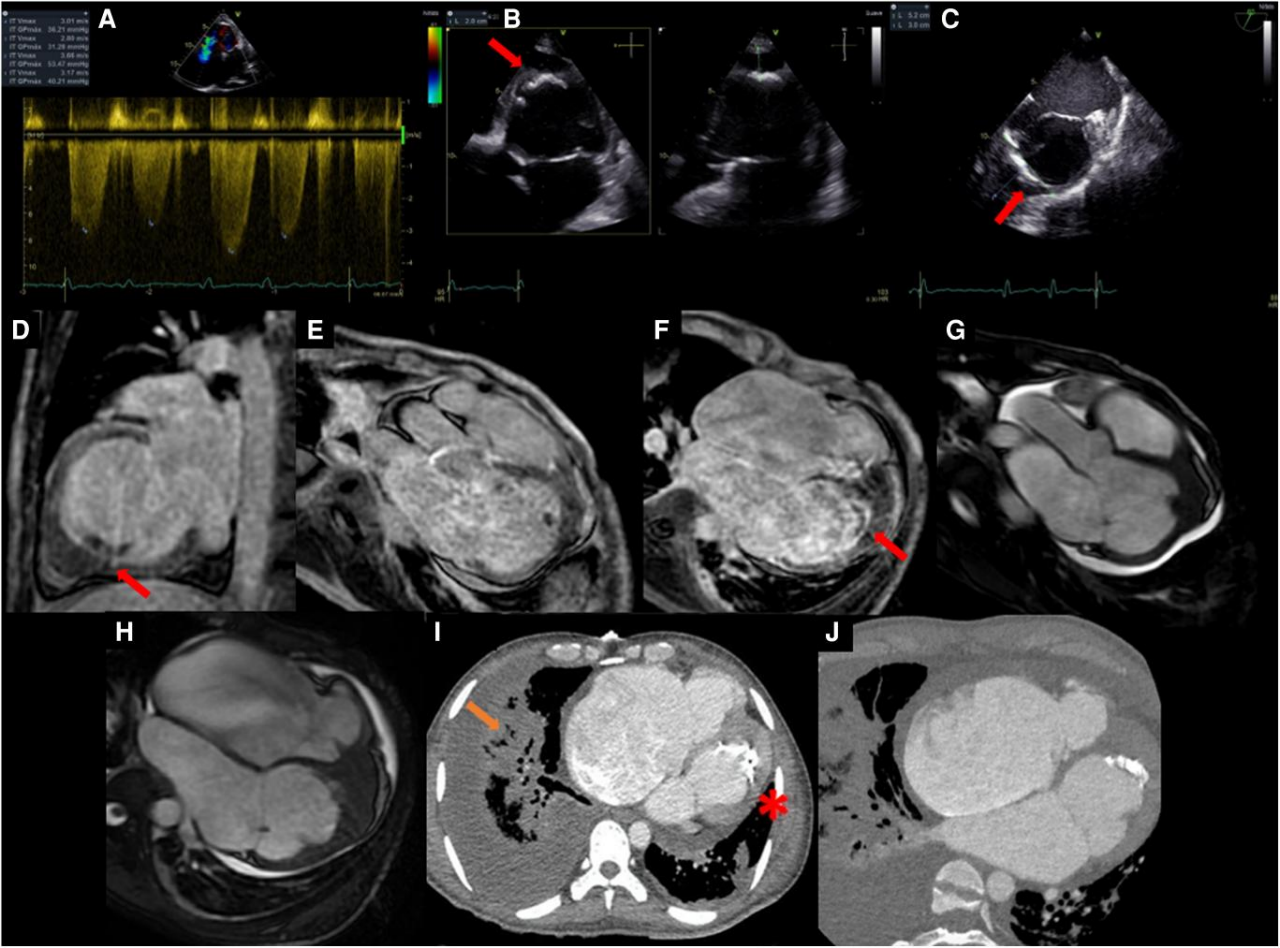
Case 2 images. (*A*) Continuous Doppler at the level of the tricuspid valve with severe regurgitation (peak velocity of 3 m/s) and septal e′ wave tissue Doppler 6 cm/s (not shown). (*B*) Echo apical four-chamber view focused on the left ventricle, and (*C*) transoesophageal echocardiography mid-oesophageal view at 60° shows calcified endocardium superimposed on apical left ventricular hypertrophy with a maximum thickness of 15 mm (red arrows). (*D–F*) Late gadolinium enhancement sequences showing the ‘double V sign’ (red arrows) with endocardial enhancement and superimposed apical thrombus. (*G*, *H*) Steady-state free precession sequence in three-chamber and four-chamber views, respectively, with significant bi-atrial dilatation and biventricular apical obliteration during diastole. (*I*, *J*) Cardiac computed tomography showing large endocardial apical calcification (asterisk) and apical obliteration with hypoattenuated image. Note the pulmonary congestion with air bronchograms (orange arrow).

A 29-year-old man was admitted to the emergency department for progressive dyspnoea, haemoptysis, palpitations, and weight loss in the last few months. There was no significant medical history. Physical examination showed cachexia with signs of peripheral congestion. Auscultation revealed tachycardia, arrhythmic heart sounds, and a mild systolic murmur in the tricuspid focus. The most relevant laboratory findings were mild eosinophilia (750 eosinophils/μL, normal range < 500 eosinophils/μL) and elevation of NT-proBNP (4514 pg/mL, normal value < 125 pg/mL) and CA-125 (526 U/mL, normal value < 35 U/mL). Electrocardiogram showed an atrial fibrillation rhythm at 120 b.p.m. Transthoracic echocardiogram showed LV apical thickening with an echodense mass obliterating the apex, and the right ventricle (RV) was dilated and dysfunctional (*Figure 2*). Computed tomography pulmonary angiogram confirmed pulmonary embolism. Computed tomography also revealed obliteration and calcification in the LV apex. He received anticoagulant and diuretic treatment, and immunological and infectious diseases such as tuberculosis were ruled out. Cardiac magnetic resonance showed obliteration and retraction of both ventricular apical regions; LGE imaging showed subendocardial enhancement in the LV with superimposed thrombus, and while the RV showed diffuse and transmural enhancement affecting the outflow tract, after analysis of these images and additional CMR sequences, it was concluded that EMF was the most likely diagnosis. Patient declined surgical treatment. During follow-up, the patient receives guideline-directed medical therapy for heart failure and full anticoagulation.

### Case 3

**Figure 3:**
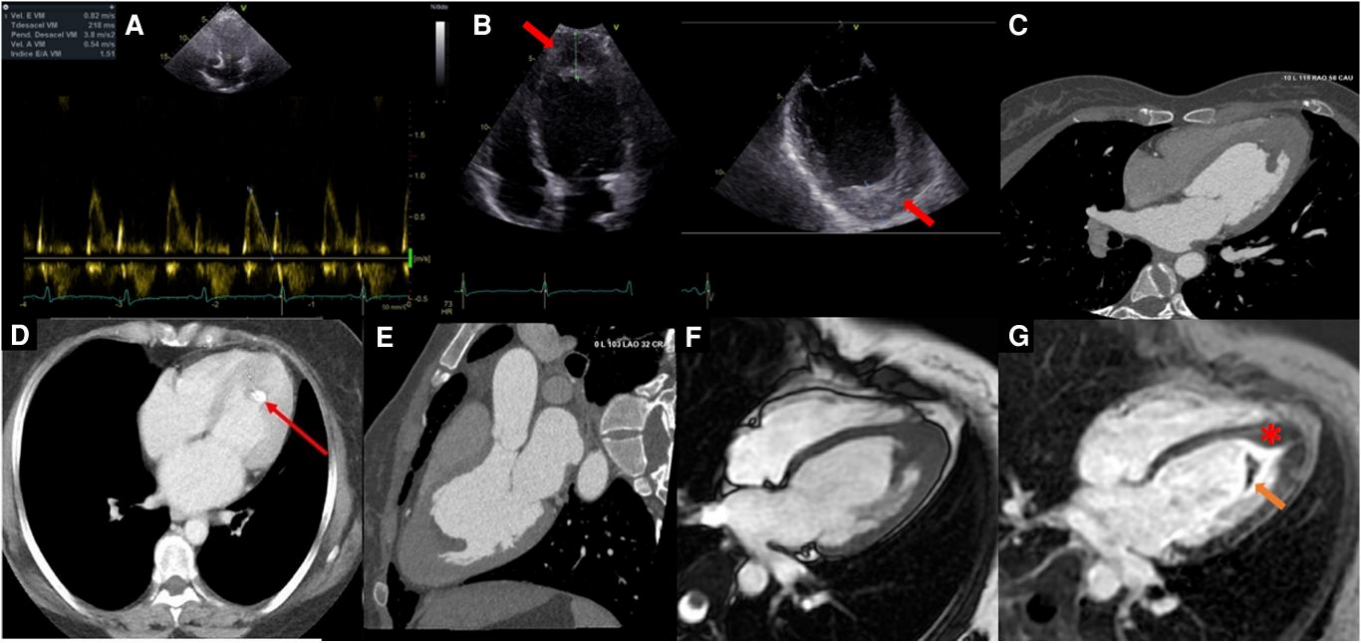
Case 3 images. (*A*) Pulsed Doppler demonstrated a restrictive filling pattern of the left ventricle and tissue Doppler with an e-wave at 4 cm/s (not shown). (*B*) Transthoracic echocardiogram. Four-chamber apical view echocardiogram with 27 mm apical thickening and endocardial calcification (red arrow). Transoesophageal echocardiography showed apical thickening and calcified areas (red arrow). (*C–E*) Cardiac computed tomography in four-chamber and three-chamber views showing apical obliteration, low attenuation LV filling defect consistent with LV thrombus, and calcification (arrow). A small left ventricular cavity is also seen. (*F*, *G*) Cardiac magnetic resonance in steady-state free precession and late gadolinium enhancement, respectively, showing apical diastolic obliteration (asterisk) and the ‘double V sign’ with endocardial enhancement and superimposed apical thrombus (orange arrow); in addition, diffuse intramyocardial enhancement is shown in the hypertrophied apical region.

A 76-year-old woman was seen for chest pain, dyspnoea, and palpitations. The patient had a history of hypertension and diabetes mellitus. She was taking bisoprolol 2.5 mg QD. Physical examination and laboratory findings were normal. Electrocardiogram showed T-wave inversion in leads I, AVL, V3–6. Transthoracic echocardiogram showed apical thickening of 27 mm with endocardial calcification and atrial dilatation with a restrictive filling pattern (*Figure 3*). Cardiac magnetic resonance evidenced predominant apical myocardial hypertrophy of 15 mm with LV apical obliteration. Late gadolinium enhancement images showed a double enhancement pattern: a first pattern of subendocardial enhancement in the apical region and a second pattern of patchy enhancement in the mid-myocardium of the LV apex, in addition to an image of superimposed thrombus. A cardiac CT scan corroborated the apical hypertrophy, showed apical endocardial calcification, and ruled out significant coronary artery disease. Due to the degree of apical hypertrophy and the two LGE patterns in this patient (unusual in EMF), it was considered that two different cardiomyopathies could be present in this patient (ApHCM-EMF). However, the genetic test was negative; there was no family history of HCM, and the pathology was unavailable; therefore, there was insufficient data to confirm a pre-existing diagnosis of ApHCM. The patient received diuretic treatment with adequate response. Subsequently, close follow-up was decided.

## Discussion

Endomyocardial fibrosis is usually prevalent in tropical regions, with an unknown aetiology; multiple factors have been associated with its development.^1^ Three phases have been described: the acute inflammatory phase with febrile syndrome, carditis, and myocardial necrosis and the subacute and chronic phases with heart failure, restrictive physiology, thromboembolic disease, and arrhythmias.^2^ Fibrosis usually extends from the apex to the posterior leaflet of the mitral valve, respecting the anterior leaflet and outflow tract, and affects the LV in 40% of cases; biventricular effects are seen in 50%.^2^

The diagnosis of this condition is frequently delayed due to its subclinical progression. Symptoms manifest depending on the affected cardiac chamber; the disease is often advanced when it becomes evident.

### Echocardiography

This ultrasound-based imaging technique is widely available and serves as the initial imaging modality when there is suspicion of this disease. However, the pathologic findings usually correspond to an advanced stage of the disease. Thus, the following echocardiographic findings have been described as typical of the disease: apical fibrosis, ventricular wall fibrosis, atrial enlargement, atrioventricular valve regurgitation, and obliteration of the ventricular cavity.^3^ Likewise, the finding (in a parasternal long-axis view) of pericardial effusion and endocardium fibrous shelf formation are very typical signs of the disease, and especially the latter can serve as a sign differential with pathologies such as rheumatic mitral valve disease.^3^ Still, nevertheless, there are signs observed in the advanced stages of the disease. Mocumbi *et al*.^4^ elaborated a series of major and minor echocardiographic criteria for diagnosis of EMF based on registries obtained in the Mozambique population, which may be applicable for screening of EMF and grading of disease severity in areas endemic for this disease. Specific technical challenges, such as inadequate tissue characterization and suboptimal apical visualization in certain patients, can hinder the diagnostic process, contributing to delays.

### Cardiac tomography

The role of CT is different in the various stages of EMF. In the first stage, unfortunately, it does not have the necessary contrast resolution to assess myocardial inflammation. In the second stage, cardiac CT helps distinguish thrombus from calcification and accurately quantifies calcified regions. This quantitative information could be important for guiding clinical and surgical decision-making in EMF patients. Additionally, it facilitates the diagnosis of associated pathologies, such as thromboembolic phenomena (as seen in Case 2). Cardiac CT has a high spatial resolution and is more accurate than CMR in studying coronary artery disease, especially when fibro/calcified plaques predominate. It can also help measure the degree of hypertrophy and demonstrate apical obliteration when echocardiography and CMR results are inconclusive. Full-beat acquisition allows ventricular and valve’s functional assessment identifying indirect signs of restriction such as small ventricular cavities and atrial enlargement, which usually occurs in the third phase.

Recently, it has been demonstrated that CT can detect fibrosis by this method, showing a strong correlation with CMR findings. However, despite a high specificity, the sensitivity is still low.^5^

### Cardiac magnetic resonance

Cardiac magnetic resonance imaging has emerged as a valuable tool in characterizing this condition due to its ability to differentiate cardiac tissues and reveal the typical patterns of involvement. Steady-state free precession sequence cine imaging allows identification of LV or RV apical obliteration associated with an enlargement of atrium characteristic of EMF, CMR-LGE technique allows accurate identification of the fibrosis pattern, and a predominant subendocardial enhancement pattern has been described in EMF, without restriction to any coronary territory, affecting mainly the apex and may extend into the inferior wall until involving the posterior portion of the mitral valve apparatus and interestingly without affecting the LV outflow tract.^6,7^

The double V sign, which consists of a three-layered pattern of normal myocardium, endocardial thickening and fibrosis, and the presence of an overlying thrombus with or without calcification in the affected ventricular apex, has been described as a typical finding of this disease. Still, however, in certain circumstances, the presence of an overlying thrombus is not present, configuring the single V sign.^6^ Acute thrombi are bright on T1 and T2 images, while subacute thrombi are more intense on T1 and less intense on T2 compared with the myocardium. In contrast, organized chronic thrombi are more challenging to identify, as they have low intensity on both images due to reduced water content and possible calcification.^7^

Cardiac magnetic resonance allows for a diagnostic approach without the need for histopathological studies, particularly since the diagnostic yield of endomyocardial biopsy remains variable, typically hovering around 50%.^6^

A predominant subendocardial enhancement pattern often characterizes EMF^7^; in contrast, ApHCM is distinguished by a heterogeneous enhancement pattern in the medial wall of the ventricle.^7,8^ The coexistence of these enhancement patterns in a patient with apical hypertrophy (as a Case 3) may lead to the tantalizing hypothesis of the presence of two rare pathologies, as previously reported.^9–12^ However, to affirm with certainty, the coexistence of these two pathologies further proof of apical HCM should be demonstrated (genetic testing demonstrating a sarcomeric pathogenic or likely pathogenic variant, endomyocardial biopsy proof of myocardial disarray, and/or other morphological features of HCM), but this is often not possible given the low prevalence of a positive genetic test (30–60%), with family history present in 60% of cases, and disorganized hypertrophy are of low prevalence in patients with HCM and even lower in its variant ApHCM,^8,12^ so we consider that there is a gap in the evidence; in some particular cases, it will be challenging to know if there really is a coexistence of both rare pathologies or perhaps midventricular hypertrophy and fibrosis might not be so infrequent and, in this context, could be secondary to EMF. The need for further studies to clarify this complex situation becomes apparent.

To sum up, EMF is a rare heart disease with a restrictive phenotype and a poor prognosis, which is characterized by apical obliteration with fibrous tissue, of one or both ventricles, and is usually associated with thrombosis, calcification, and atrioventricular valve insufficiency. Multimodality imaging is essential for the initial and definitive diagnosis of this disease and to distinguish this pathology from others. Transthoracic echocardiogram and transoesophageal echocardiography are the first-line imaging modalities to assess for EMF, whereas CMR allows confirmation of the diagnosis by identifying the ‘double V’ sign, which is pathognomonic of EMF.

## Supplementary Material

ytaf039_Supplementary_Data

## Data Availability

We declare that ‘No new data were generated or analysed in support of this research’.

## Lead author biography

Dr Josh Moscoso is a Latin American physician working as a clinical cardiologist at the Guillermo Almenara Hospital in Lima, Peru. He has a strong interest in structural heart disease and cardiovascular interventional cardiology.

## Supplementary material

**Consent:** We declare that ‘Complete written informed consent, complying with COPE guidelines, was obtained from the three patients for the publication of this study and accompanying images’.

**Funding:** We declare that no financial support was received for the research, authorship, and/or publication of this article.
